# Genome Wide Association Mapping of Grain Arsenic, Copper, Molybdenum and Zinc in Rice (*Oryza sativa* L.) Grown at Four International Field Sites

**DOI:** 10.1371/journal.pone.0089685

**Published:** 2014-02-25

**Authors:** Gareth J. Norton, Alex Douglas, Brett Lahner, Elena Yakubova, Mary Lou Guerinot, Shannon R. M. Pinson, Lee Tarpley, Georgia C. Eizenga, Steve P. McGrath, Fang-Jie Zhao, M. Rafiqul Islam, Shofiqul Islam, Guilan Duan, Yongguan Zhu, David E. Salt, Andrew A. Meharg, Adam H. Price

**Affiliations:** 1 Institute of Biological and Environmental Sciences, University of Aberdeen, Aberdeen, United Kingdom; 2 Department of Horticulture and Landscape Architecture, Purdue University, West Lafayette, Indiana, United States of America; 3 Department of Biological Sciences, Dartmouth College, Hanover, New Hampshire, United States of America; 4 USDA ARS, Dale Bumpers National Rice Research Center, Stuttgart, Arkansas, United States of America; 5 Texas A&M University System, Texas A&M AgriLife Research, Beaumont, Texas, United States of America; 6 Rothamsted Research, Harpenden, Hertfordshire, United Kingdom; 7 College of Resources and Environmental Sciences, Nanjing Agricultural University, Nanjing, China; 8 Department of Soil Science, Bangladesh Agricultural University, Mymensingh, Bangladesh; 9 Research Center for Eco-environmental Sciences, Chinese Academy of Sciences, Beijing, China; 10 Institute for Global Food Security, Queen’s University Belfast, David Keir Building, Belfast, United Kingdom; International Rice Research Institute, Philippines

## Abstract

The mineral concentrations in cereals are important for human health, especially for individuals who consume a cereal subsistence diet. A number of elements, such as zinc, are required within the diet, while some elements are toxic to humans, for example arsenic. In this study we carry out genome-wide association (GWA) mapping of grain concentrations of arsenic, copper, molybdenum and zinc in brown rice using an established rice diversity panel of ∼300 accessions and 36.9 k single nucleotide polymorphisms (SNPs). The study was performed across five environments: one field site in Bangladesh, one in China and two in the US, with one of the US sites repeated over two years. GWA mapping on the whole dataset and on separate subpopulations of rice revealed a large number of loci significantly associated with variation in grain arsenic, copper, molybdenum and zinc. Seventeen of these loci were detected in data obtained from grain cultivated in more than one field location, and six co-localise with previously identified quantitative trait loci. Additionally, a number of candidate genes for the uptake or transport of these elements were located near significantly associated SNPs (within 200 kb, the estimated global linkage disequilibrium previously employed in this rice panel). This analysis highlights a number of genomic regions and candidate genes for further analysis as well as the challenges faced when mapping environmentally-variable traits in a highly genetically structured diversity panel.

## Introduction

Rice (*Oryza sativa* L.) is perhaps the most important crop plant given that it is estimated to be the staple food of half the world’s human population. As this population rises over the coming decades the demand for staple crops, like rice, will grow further and increasing crop yield while maintaining grain quality is, therefore, essential. For people who are reliant on cereals as a dietary staple, a significant proportion of their minerals come from these grains. Improved concentrations of essential micro nutrients such as zinc, and reductions in the potentially toxic trace elements such as arsenic would therefore hold significant potential for improvements in human health [1], [2].

There is significant genetic diversity in rice which can be exploited to perform genome-wide association (GWA) mapping, allowing the unravelling of complex physiological traits [3]–[6]. Rice landraces and cultivars have undergone many recombination events compared to experimental mapping populations. GWA mapping takes advantage of this increased recombination to potentially localize the genetic determinants of traits to narrower regions in the genome. However, populations used for GWA studies can have a low power to detect rare alleles unless the population is large. This contrasts with biparental experimental mapping populations in which all alleles are generally at frequencies of 25–50% (depending on the population type and the heterozygosity of the parents). Populations of rice have been developed to exploit the power of GWA mapping. The Rice Diversity Panel 1 (RDP1) of 421 accessions genotyped with 36,901 high quality single nucleotide polymorphisms (SNPs) [7]–[9] has been used to identify genomic regions associated with flowering time, panicles per plant, seed number per panicle, seed morphology traits, blast resistance, amylose content, protein content [5] and aluminium tolerance [4]. A different rice panel has been developed from 517 Chinese landraces [3]. In that study GWA mapping was performed on 14 agronomic traits across 373 *indica* accessions using 671,355 common SNPs. Traits studied included tiller number, grain morphology traits, amylose content, heading date, and drought tolerance [3]. More recently, the same group increased their study population to 950 cultivars including cultivars from both the *Indica* and *Japonica* subspecies [6]. Using this population Huang et al. [6] were able to identify a further 32 loci associated with flowering time, and 10 grain related traits. The authors concluded from this study that the additional sample size increased the power of detection for trait-associated DNA variants using GWA mapping [6].

Improvement in the content of the micro nutrient zinc within rice grains could have a positive impact on human health [1] since conservative estimates suggest that greater than 25% of the world’s population are at risk from zinc deficiency [10]–[12]. There is genetic variation amongst rice cultivars for grain zinc concentration, and a number of quantitative trait loci (QTLs) have also been identified in rice [13]–[22].

Unlike zinc, there is not a widespread global issue with copper deficiency in humans [23]. However, copper is an essential element for plants and has many functions including having a role in the electron transport chain of photosystem I, acting as a component of some metalloproteins and as a co-factor in a number of enzymes including cytochrome c oxidase, ascobate oxidase and amino oxidase [24].

Elevated inorganic arsenic concentration in rice grains has been identified as a risk to human health where rice is a dietary staple [25]–[29]. Inorganic arsenic is a non-threshold class 1 human carcinogen [30]. It has been established that there is genetic variation for grain arsenic accumulation [2], [31]–[35], and QTLs have been identified [17], [21], [22], [36]. However, grain arsenic concentrations are also strongly affected by the environment [31], [34], [35].

Molybdenum is an essential element required for plants and animals as a component of the molybdopterin cofactor in a number of enzymes including nitrate reductase, sulfite oxidase, xanthine dehydrogenase, aldehyde oxidase and mitochondrial amidoxime reductase [37]. Also, grain molybdenum may be important for seedling vigour in acid soils with low molybdenum bioavailability. A number of genes have been identified as potential transporters of molybdenum in plants [37], including *MOT1* in *Arabidopsis thaliana* where natural variation in this gene is responsible for variations in the accumulation of molybdenum in shoots and roots [38]–[40].

To date GWA mapping has not been used for the exploration of loci controlling accumulation of essential minerals and potentially toxic trace elements in rice grain. Here, we report such a study, using the RDP1 to identify a large number of SNPs associated with grain accumulation of zinc, copper, molybdenum and arsenic in rice grains for plants grown in four different field locations. The stability of these QTLs is explored across years and between different environmental conditions, as well as their co-localisation with previously identified QTLs and candidate genes.

## Results and Discussion

### Variation in Grain Elemental Concentrations

At the Faridpur field site, over 50% of the variation for each element in grain (arsenic, copper, molybdenum, and zinc) is explained by differences between cultivars (Table 1). For the traits at the Qiyang field site, grain molybdenum was the only trait where over 50% of the observed variation could be explained by cultivar. For grain arsenic and zinc the percentage of variation explained by cultivar was around 40%, while grain copper was approximately 20%. For the Arkansas field site (both 2006 and 2007) over 40% of the variation for each element could be explained by differences between cultivars. At the Texas field site the cultivars explain greater than 39% of the variation for the four elements. At all sites, for all four elements measured there were significant differences between the rice subpopulations (Table 1). Full details of the differences between subpopulations are given in Table S1. General trends for the subgroups were that *aus* tend to be highest in grain arsenic while either *tropical* or *temperate japonicas* were the lowest, the opposite is true for grain copper, *indicas* were the lowest in zinc and no clear pattern across sites exists for grain molybdenum.

**Table 1:** Mean and range of grain element concentrations.

Site	Trait	No of cultivars	Descriptive statistics for cultivars				F-value for cultivar (all significant at P<0.001)	Proportion of thevariation explainedby cultivar (%)	F-value for subpopulation (all significant at P<0.001)^1^
			Mean	Min.	Median	Max.			
Faridpur	Grain As (mg kg^−1^)^2^	312	0.443	0.192	0.435	0.899	7.77	63.4	12.5
Faridpur	Grain Cu (mg kg^−1^)	312	3.767	1.96	3.66	7.46	5.33	52.5	48.1
Faridpur	Gain Mo (mg kg^−1^)	312	1.030	0.556	0.978	2.088	7.43	62.2	8.27
Faridpur	Grain Zn (mg kg^−1^)	312	17.022	10.32	16.19	32.97	9.26	67.8	41.0
Qiyang	Grain As (mg kg^−1^)^2^	295	0.675	0.363	0.662	1.266	3.58	40.1	42.0
Qiyang	Grain Cu (mg kg^−1^)	295	0.755	0.274	0.662	3.338	2.00	20.5	7.6
Qiyang	Grain Mo (mg kg^−1^)	295	1.554	0.842	1.521	3.958	7.03	61.0	11.7
Qiyang	Grain Zn (mg kg^−1^)	295	16.518	7.155	16.394	33.977	3.59	40.2	19.6
Ark2006	Grain As (mg kg^−1^)^2^	336	0.375	0.100	0.359	0.988	4.18	41.0	15.29
Ark2006	Grain Cu (mg kg^−1^)	336	2.241	0.972	2.139	4.631	6.69	55.5	21.4
Ark2006	Grain Mo (mg kg^−1^)	336	0.708	0.364	0.704	1.412	12.6	71.8	20.17
Ark2006	Grain Zn (mg kg^−1^)	336	25.301	17.918	25.483	35.476	8.03	60.6	51.47
Ark2007	Grain As (mg kg^−1^)^2^	348	0.256	0.030	0.205	1.400	14.02	70.7	15.83
Ark2007	Grain Cu (mg kg^−1^)	348	3.736	1.989	3.725	6.224	6.7	51.5	30.36
Ark2007	Grain Mo (mg kg^−1^)	348	0.458	0.139	0.420	1.194	16.34	74.0	51.79
Ark2007	Grain Zn (mg kg^−1^)	348	30.017	20.844	29.734	42.410	7.8	55.8	44.10
Tx2009	Grain As (mg kg^−1^)^2^	370	0.628	0.172	0.618	1.682	5.09	58.2	11.46
Tx2009	Grain Cu (mg kg^−1^)	370	2.972	1.655	2.907	5.497	9.5	74.3	61.34
Tx2009	Grain Mo (mg kg^−1^)	370	0.343	0.116	0.337	0.730	14.1	81.6	23.35
Tx2009	Grain Zn (mg kg^−1^)	370	21.513	11.938	21.538	40.021	2.92	39.5	20.77

1 Subpopulation group analysis was performed using the allocation of the cultivars to the four subpopulation groups: *aus*, *indica*, *temperate japonica* and *tropical japonica*. Mean values for the subpopulation groups are presented in Table S1.

2 This (summarised) data has previously been reported in Norton et al. (35).

Analysis of cultivar differences and subpopulation group differences are presented as the F value from two separate one-way ANOVA tests, one for cultivar and one for subpopulation.

Previously using the grain arsenic data for each of the cultivars it was possible to identify cultivars that have low grain arsenic across multiple field sites [35]. Three cultivars were identified, all *temperate japonicas*, that have low grain arsenic across four of the five field sites [35]. These cultivars could be introduced into breeding programs with the goal of breeding for low grain arsenic rice cultivars. The breeding of low grain arsenic rice cultivars could have an impact on the amount of arsenic that is being consumed from rice in countries like Bangladesh.

An increase in rice grain zinc could have an impact on human health [1] for the estimated 25% of the world population that are at risk from zinc deficiency [10]–[12]. From this study we have identified five cultivars that have high grain zinc (in the top 20% of all the cultivars common across all five field trials) at each of the field trials (Table 2). These are Estrela (admix), Bulgare (*temperate japonica*), Jamir (*aus*), Khao Tot Long 227 (*aus*) and DZ 193 (*aus*). These cultivars have the potential to be exploited in rice breeding programs to increase rice grain zinc, therefore addressing human zinc deficiency.

**Table 2:** Cultivars which were in either the upper (high grain zinc) or lower (low grain zinc) 20% in all of the five field trials.

Cultivar name	Subgroup	Grain zinc concentration
Gharib	*indica*	Low
JC 117	*indica*	Low
LD 24	*indica*	Low
Bulgare	*temperate japonica*	High
DZ 193	*aus*	High
Estrela	admix	High
Jamir	*aus*	High
Khao Tot Long 227	*aus*	High

The value is based on the mean grain zinc value in the cultivars which are common across the five trials.

### Genome Wide Association Mapping of Grain Arsenic, Copper, Molybdenum and Zinc

**Figure 1:**
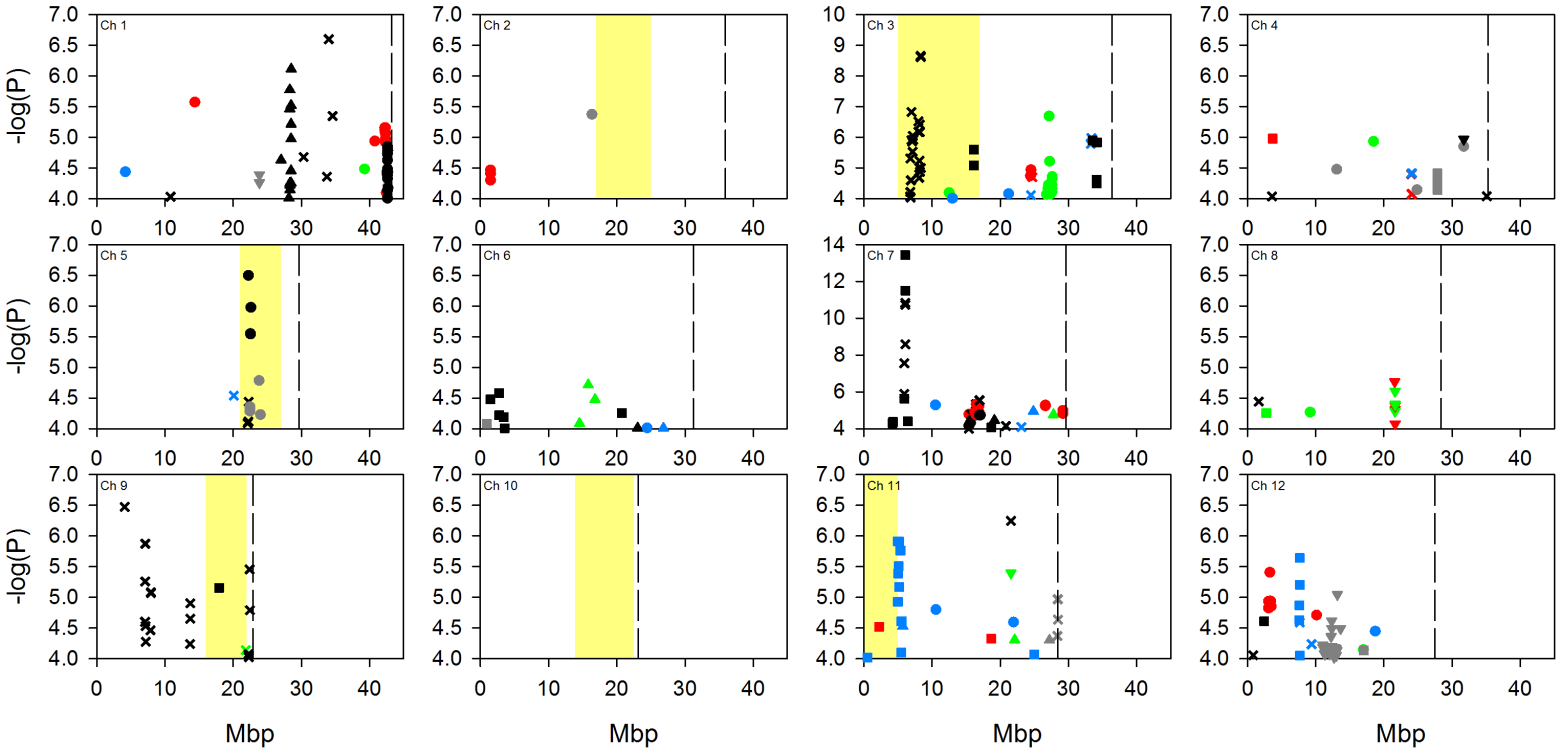
GWA mapping of grain arsenic concentration at the five field sites across the 12 rice chromosomes. Data points represent SNPs significantly associated (p<0.0001) with the trait and which have a MAF >5%. Significant SNPs from different experiments are displayed with different coloured symbols: 2006 Arkansas are red, 2007 Arkansas are black, 2009 Texas are blue, Faridpur are green, and Qiyang are grey. Analyses of the combined subpopulation groups and separate subpopulations are represented by different symbols: combined analysis = X, *aus* = circle, *indica* = square, *tropical japonica* = triangle, *temperate japonica* = inverted triangle. Yellow highlighted bars indicate regions of mapped QTLs for grain arsenic concentration (21, 22). Dotted lines indicate chromosome ends.

**Figure 2:**
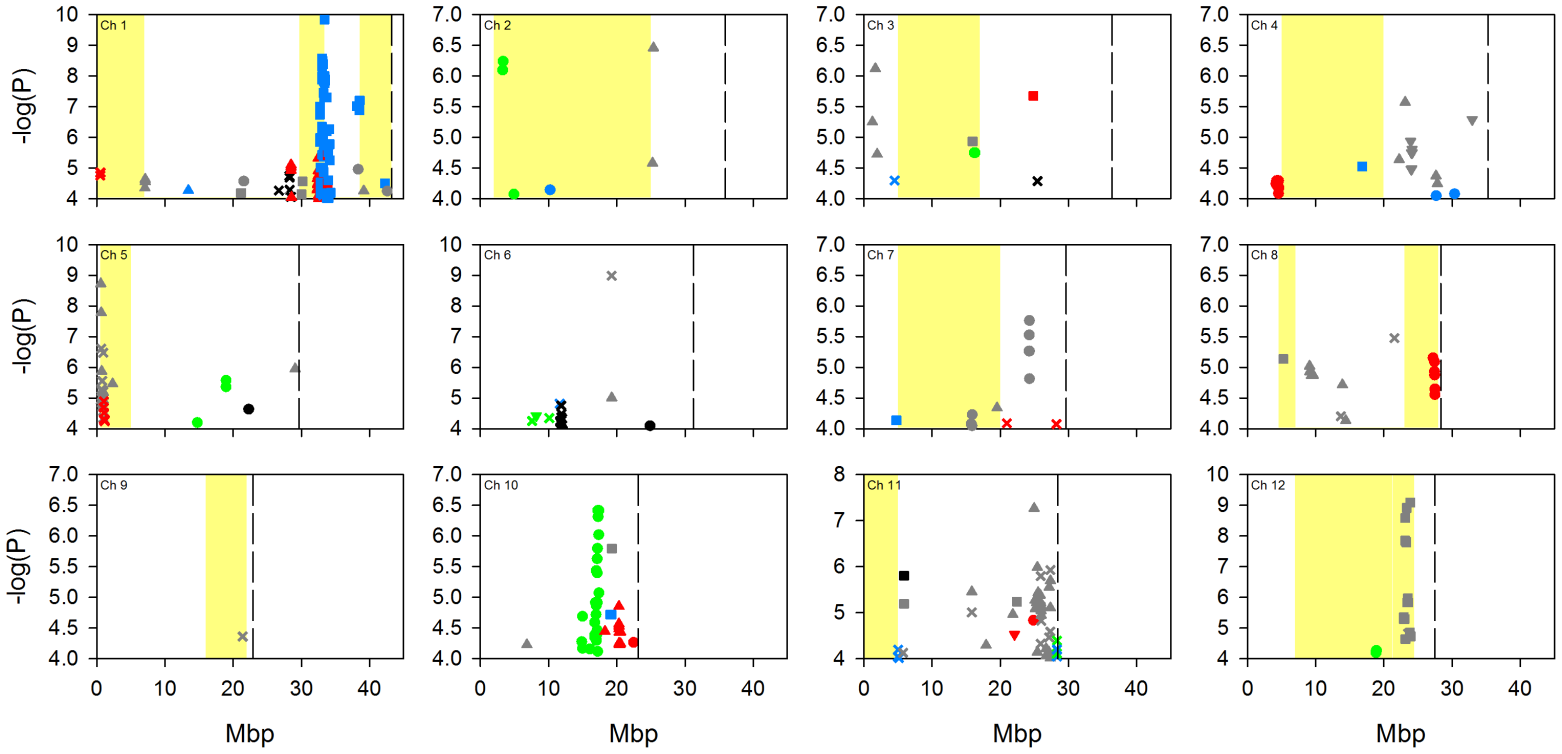
GWA mapping of copper concentration at the five field sites across the 12 rice chromosomes. Data points represent SNPs significantly associated (p<0.0001) with the trait and which have a MAF >5%. Significant SNPs from different experiments are displayed with different coloured symbols: 2006 Arkansas are red, 2007 Arkansas are black, 2009 Texas are blue, Faridpur are green, and Qiyang are grey. Analyses of the combined subpopulation groups and separate subpopulations are represented by different symbols: combined analysis = X, *aus* = circle, *indica* = square, *tropical japonica* = triangle, *temperate japonica* = inverted triangle. Yellow highlighted bars indicate regions of mapped QTLs for grain copper concentration (17, 21, 22). Dotted lines indicate chromosome ends.

**Figure 3:**
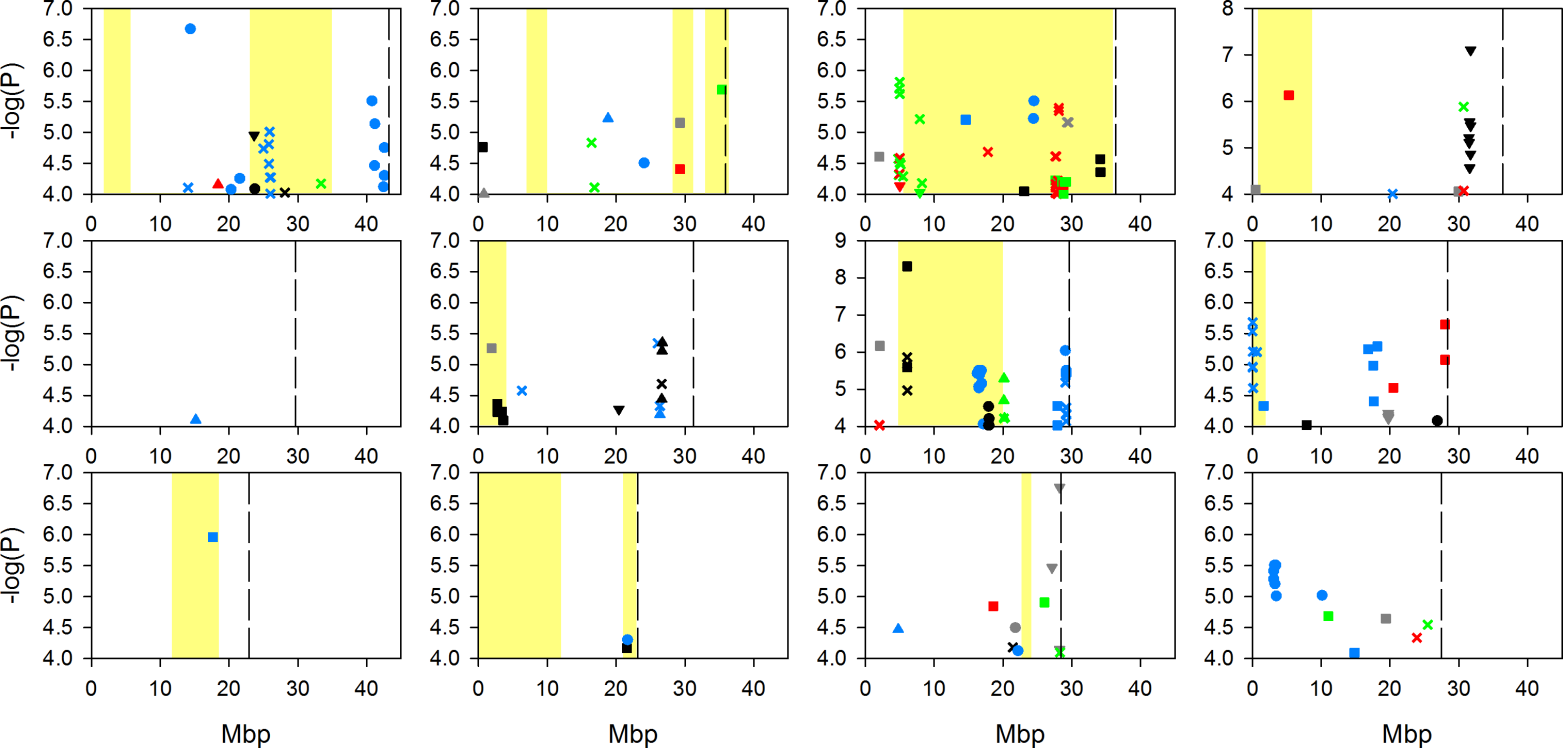
GWA mapping of molybdenum concentration at the five field sites across the 12 rice chromosomes. Data points represent SNPs significantly associated (p<0.0001) with the trait and which have a MAF >5%. Significant SNPs from different experiments are displayed with different coloured symbols: 2006 Arkansas are red, 2007 Arkansas are black, 2009 Texas are blue, Faridpur are green, and Qiyang are grey. Analyses of the combined subpopulation groups and separate subpopulations are represented by different symbols: combined analysi = Xs, *aus* = circle, *indica* = square, *tropical japonica* = triangle, *temperate japonica* = inverted triangle. Yellow highlighted bars indicate regions of mapped QTLs for grain molybdenum concentration (17, 21, 22). Dotted lines indicate chromosome ends.

**Figure 4:**
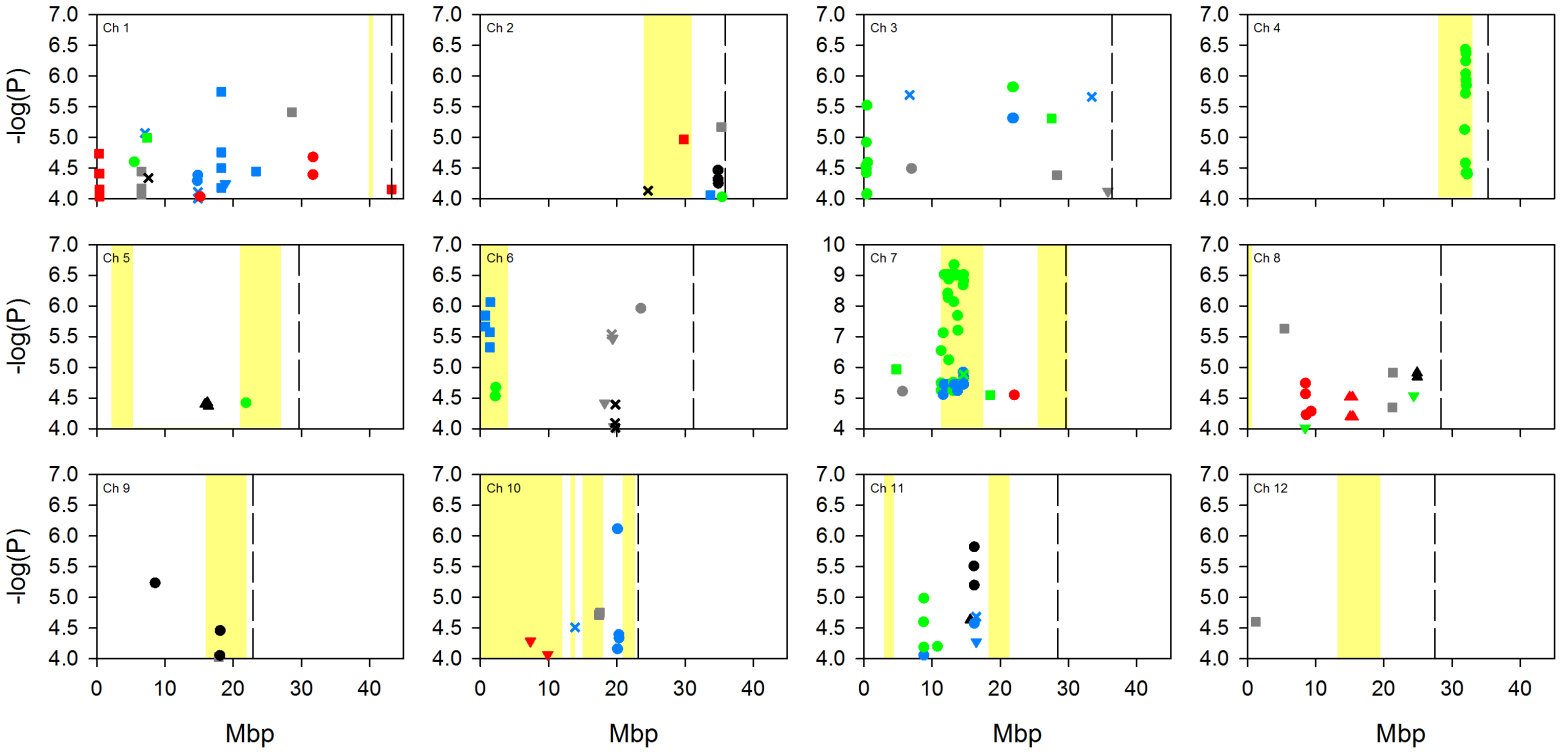
GWA mapping of zinc concentration at the five field sites across the 12 rice chromosomes. Data points represent SNPs significantly associated (p<0.0001) with the trait and which have a MAF >5%. Significant SNPs from different experiments are displayed with different coloured symbols: 2006 Arkansas are red, 2007 Arkansas are black, 2009 Texas are blue, Faridpur are green, and Qiyang are grey. Analyses of the combined subpopulation groups and separate subpopulations are represented by different symbols: combined analysis = X, *aus* = circle, *indica* = square, *tropical japonica* = triangle, *temperate japonica* = inverted triangle. Yellow highlighted bars indicate regions of mapped QTLs for grain zinc concentration (15–17, 20, 21, 22). Dotted lines indicate chromosome ends.

Due to the complex population structure within rice, there is no single design or analysis method which will sufficiently disentangle the genetics underlying complex polygenic traits [5]. To address the complexity of population structure in this analysis, GWA mapping was performed on a worldwide population (denoted “all” below) using a mixed model to account for the population structure, and was also performed within four separate subpopulations (*aus*, *indica*, *temperate japonica* and *tropical japonica*) as defined by Zhao et al. [5]. A naïve approach was not conducted due to the high potential of false discoveries [5]. Using both GWA mapping approaches we identified a large number of SNPs significantly associated with the traits (Figures 1–4; full list of SNPs are detailed in Tables S3, S4, S5 and S6). Figures 1–4 indicate the location of SNPs with a P-value below the assigned significance threshold P<0.0001 associated with the traits. Significant SNPs from the different experiments are displayed in different coloured symbols and the analysis of the combined subgroups and separate subgroups are represented by different symbols. Also presented in these figures are the positions of previously identified QTLs.

### Identification of Quantitative Trait Loci Common Across Multiple Environments

As the population was grown across multiple sites, the stability of the QTLs across multiple environments was explored. As described in the methods section, a SNP was called significant if the P-value was <0.0001 and with a minor allele frequency (MAF) >5%. The significant SNPs had to be within 200 kb of each other to be considered close enough to be the same genomic region. A value of 200 kb was selected as this was the estimated global linkage disequilibrium (LD) previously employed in this rice population [5]. There were a total of 17 chromosomal regions where significant SNPs were associated with a trait (e.g., arsenic) within one or more analyses across two field experiments (Figures 1–4). For example, with grain copper significant SNPs at the top of chromosome 5 at approximately 0.95 Mb, were associated with the analysis of “all” accessions and the *tropical japonica* subpopulation at the Qiyang field site, and in the same region in “all” accessions at the Arkansas field site in 2006 SNPs. Of these 17 locations, six of them were located with a previously detected QTL [17], [22]. Details of these six locations are presented in Table 3; one of them is for arsenic, two for copper, one for molybdenum and two for zinc. Due to the uncertainty of the mapping position of the previously detected QTLs, the physical range they cover is quite large. This means that there is a chance that the co-localisation of SNPs within a previously identified QTL region may be down to chance rather than co-localisation.

**Table 3:** Co-localisation of significant SNPs from multiple field experiments detected in this study and previously detected QTLs.

Trait	Chromosome	Positionof SNPs (Mb)	GWA mapping experiments where significant SNP(s) were detected	Population in which QTLs were detected in same region
As	5	∼22.3	Arkansas 2007 (“all” and *aus*); Qiyang (*aus*)	Lemont×TeQing RIL (22)
Cu	1	∼32.6	Arkansas 2006 (*tropical japonica*); Texas (*indica*)	Bala×Azucena (17)
Cu	5	∼0.9	Arkansas 2006 (“all”); Qiyang (“all” and *tropical japonica*)	Lemont×TeQing TIL (22)
Mo	10	∼21.5	Arkansas 2007 (*indica*); Texas (*aus*)	Lemont×TeQing TIL (22)
Zn	7	11.3–14.6	Faridpur (“all” and *aus*); Texas (*aus*)	Bala×Azucena (17)
Zn	9	∼18.0	Arkansas 2007 (*aus*); Qiyang (*indica*)	Lemont×TeQing TIL (22)

The arsenic association localises with a previously detected QTL on chromosome 5 originally identified in a ‘Lemont’ × ‘TeQing’ recombinant inbred line (RIL) mapping population ([22]; Lemont is a *tropical japonica* cultivar from the USA and TeQing is an *indica* cultivar from China). In the current study, the significant arsenic association was detected in the *aus* subpopulation and “all” accessions in the Arkansas 2007 field site, and the *aus* subpopulation at the Qiyang field site. One of the copper associations is on chromosome 1 where significant SNPs from the *tropical japonica* subpopulation GWA mapping from the Arkansas 2006 field site and the *indica* subpopulation GWA mapping from the Texas field site co-localise with a QTL detected in the ‘Bala’ (derived from a cross between an *indica* and *aus* cultivar) × ‘Azucena’ (*tropical japonica*) mapping population [17]. The second copper association which localises with a QTL on chromosome 5, where significant SNPs were detected in both the “all” accessions analysis and the *tropical japonica* subpopulation at the Qiyang field site and the “all” accessions analysis at the Arkansas 2006 field site. This QTL for copper was previously detected in the population of TeQing-into-Lemont introgression lines (TILs; [22]).

The significant SNPs which were associated with grain molybdenum were in the same genomic region as a previously identified QTL on chromosome 10. The SNPs were detected in the *aus* subpopulation in Texas and the *indica* subpopulation at the Arkansas field site in 2007, while the QTL was detected in the Lemont × TeQing TIL population [22].

For zinc, significant SNPs on chromosome 9 were associated with grain zinc in the *indica* subpopulation at the Qiyang field site and in the *aus* subpopulation in the Arkansas field site in 2007. In the Lemont × TeQing TIL population a QTL had previously been detected in this same region [22]. On chromosome 7 between 11.3 Mb to 14.6 Mb there were a large number of SNPs associated with grain zinc. SNPs in this region were from the “all” accessions analysis and the *aus* subpopulation analysis at Faridpur and the *aus* subpopulation at the Texas field site. These SNPs localise with previously identified grain zinc QTLs [17]. What makes this region of added interest is that it spans such a large distance, 3.3 Mb, approximately 16 times greater than the LD distance proposed by Zhao et al. for the whole population [5]. The significant SNPs that span this region were from the *aus* subpopulation analysis (SNPs from the “all” accessions population analysis were only at ∼14.6 Mb). This would indicate that either LD is greater for the *aus* subpopulation within this region or that this region contains multiple QTLs for grain zinc. Previously, it was demonstrated that LD varied for the different rice subpopulations [3], [41], but in none of the subpopulations was the LD 3 Mb. It has also been shown that rice LD is variable depending on the genomic region [3]. An explanation of why LD could be larger in this region is that this region was under recent selection within the *aus*. In *A. thaliana* it was demonstrated that LD is 10 times greater around the FLOWERING LOCUS C (FLC) compared to the whole genome LD [42]. The FLC contains the vernalization-response gene FRIGIDA (FRI) with the FRI gene undergoing a recent positive selection [43].

Also of note is the observation, clearly seen in Figures 1–4, that there were a number of associations that were only detected in a single location/year analysis that co-localise with previously detected QTLs (Figures 1–4). For brevity, these are not discussed individually here.

### Identification of Quantitative Trait Loci Common Across Years

As well as addressing environmental stability of QTLs, the year-to-year stability of QTLs was explored. In 2006 and 2007 the panel was grown at the same site in Arkansas, using a nearby field area (equivalent soil) and identical field management practices. The correlations of the traits across both years are presented in Figure S1 revealing high significance (P>0.001) and r values of 0.407 copper; 0.407 arsenic; 0.555 molybdenum and 0.630 zinc. There is only one region where SNPs with a P-value below the threshold of 0.0001 were present for the same trait across both years. This was on chromosome 1 at 42 Mb where there were SNPs associated with arsenic within the *aus* subpopulation. The lack of year-to-year stability for QTLs is unexpected, as it could be predicted that the largest driving forces for the accumulation of different concentrations of elements within grains would be soil chemistry which should vary relatively little as the plants were grown in nearby field areas (equivalent soil type) using essentially identical field management practices in two consecutive years. However, if other factors like grain yield and flower time have an effect on the accumulation of the elements in the grain then this year-year variation in QTLs may not be unexpected. It has been shown that QTLs for both yield and flowering time can be either stable or unstable across years [44], [45]. QTL stability is discussed further below.

### Identification of Common Significant SNPs using Enrichment Analysis

A second method of determining if the same genomic regions have a significant effect on the accumulation of elements across different field experiments was performed. For this method the 1% most highly significant SNPs (∼369 SNPs) for each trait separately (e.g., arsenic), based on p-value, were compared across all the GWA studies analysed per individual location. This analysis was performed on the GWA studies only across “all” accessions analysis and the 1% most significant SNPs were selected regardless of the minor allele frequency (MAF) value. Where a SNP was detected in four of the five sites it is reported in Table 4. There were no individual SNPs present in the top 1% for more than three of the five GWA studies for copper. For arsenic and zinc GWA studies there was only a single SNP present in the top 1% in four of the five site analyses at 24.69 Mb on chromosome 3 and 22.26 Mb on chromosome 3, respectively. However, for molybdenum GWA mapping there were 13 SNPs that were present in the top 1% for four site analyses, and one present in all GWA studies. A number of these molybdenum-associated SNPs were within 200 kb of each other. For example, on chromosome 3 there were four significant SNPs between 27.63–27.67 Mb.

**Table 4:** SNPs identified in GWA studies as being in the top 1% most significant in at least four of the five field sites, with site identified with Y.

				Field Site				
Trait	Ch.	Mb	SNP	ARK06	ARK07	TX09	FAR	QIY
As	3	24.69	ud3001374	Y	–	Y	Y	Y
Mo	2	29.29	id2012831	Y	–	Y	Y	Y
Mo	3	27.63	id3012036	Y	–	Y	Y	Y
Mo	3	27.67	dd3000580	Y	–	Y	Y	Y
Mo	3	27.67	id3012159	Y	–	Y	Y	Y
Mo	3	27.67	id3012161	Y	Y	Y	Y	Y
Mo	3	28.11	id3013000	Y	–	Y	Y	Y
Mo	3	28.12	id3013065	Y	–	Y	Y	Y
Mo	4	5.30	id4002236	Y	–	Y	Y	Y
Mo	4	29.85	id4010041	Y	–	Y	Y	Y
Mo	4	30.69	id4010426	Y	Y	–	Y	Y
Mo	5	15.70	id5006313	Y	–	Y	Y	Y
Mo	8	0.09	id8000032	Y	Y	Y	–	Y
Mo	8	0.17	wd8000030	Y	Y	Y	–	Y
Mo	8	0.65	id8000230	Y	Y	Y	Y	–
Zn	3	22.26	id3010343	Y	–	Y	Y	Y

### Candidate Genes Near Associated SNPs

For the elements measured in the rice grains in this study, there is varying knowledge on the molecular mechanisms of their uptake, transport and accumulation in plants. To test if candidate genes involved in the uptake, transportation or accumulation of these mineral elements were located near associated SNPs, two approaches were taken. For arsenic, copper and zinc, a small subset of candidate genes were tested, with all these genes having a known function for transporting these elements in rice. The transporters of molybdenum are unknown in rice, but transporters of molybdenum have been identified in other plant species [37], therefore the rice orthologues of these genes were tested to see if they were associated with significant SNPs detected in this study.

For arsenic transport in rice, *Lsi1* (a NIP type aquaporin) and *Lsi2* (a silicon/arsenite efflux carrier) have been identified as inter and extra cellular transporters of arsenic [46]. The gene encoding *LSI1* is located on chromosome 2 at 31266245–31269960 bp (LOC Os02g51110) (Rice Genome Annotation Project (RGAP) (http://rice.plantbiology.msu.edu)), whereas the gene encoding *LSI2* is located on chromosome 3 at 432878-430478 bp (LOC_Os03g01700). There were no significant SNPs associated with grain arsenic within 200 kb of either of these two genes.

For molybdenum there are four known transporter genes in plants [37], *MOT1* and its homologue *MOT2* in *A. thaliana*, plus *SHST1* in *Stylosanthes hamata* and *MOT2* (not a homologue of *A. thaliana* MOT1) from *Chlamydomonas reinhardtii* [39], [47]–[49]. The closest rice orthologue(s) for these four known transporter genes were identified using BLASTp analysis against the RGAP as describe in the methods section. For a number of these orthologues there are significant SNPs within 200 kb of the candidate genes (Figure 3) as described in the paragraph below. For the *MOT1* orthologue on chromosome 8 (LOC_Os08g01120) there were significant SNPs for grain molybdenum detected at the Texas field site within the “all” accessions analysis. Also of note, a QTL in this region was identified in a Bala × Azucena mapping population [21] as well as within two Lemont × TeQing progeny populations [22]. For the highest BLASTp hit of the *SHST1* gene on rice chromosome 3 (LOC_Os03g09970), there were significant SNPs associated with grain molybdenum concentration within 200 kb of the orthologue. The SNPs that were significant at this location were detected within the “all” accessions analysis at the Faridpur field site and within the “all” accessions analysis and *temperate japonicas* subpopulation analysis at the Arkansas field site in 2006. The other two *SHST1* orthologues in rice were not near significant SNPs. For the *A. thaliana MOT2* orthologue on rice chromosome 1 (LOC_Os01g45830) there were significant SNPs detected within 200 kb for the Texas “all” accessions analysis, while for *MOT2* from *C. reinhardtii* there were no SNPs significantly associated with grain molybdenum for either of the two rice orthologues. The location of significant SNPs associated with grain molybdenum at three of the four known plant molybdenum transporters suggests these genes are likely rice molybdenum transporters.

There are a number of genes (and gene families) that have been identified as being involved in the uptake, transport, and accumulation of copper and zinc in plants (for review see White and Broadley; [50]). These include, but are not limited to, the *ZIP* (Zinc-regulated transporter (ZRT), Iron-regulated transporter (IRT)-like protein) family, *YSL* (yellow stripe-like) proteins, *HMA* (heavy metal transporting ATPase) family, *MTP*s (metal tolerance proteins), *COPT* (COPper Transporter)/Ctr (Copper transporter) family, metallotheioneins, copper chaperone proteins, and *NRAMP*s (Natural Resistance Associated Macrophage Proteins). Some of these gene families have been implicated in the transport of copper and zinc as well as other elements such as iron, magnesium and cadmium. As there are such a large number of genes implicated in the transport of copper and zinc in plants, we limited our analysis to only those that have been directly identified as having a role in the uptake, transport, and accumulation of these elements in rice. For zinc, six *ZIP* genes (*ZIP*1, 3, 4, 5, 7a and 8) have all been identified as being directly involved in rice zinc transport [23], [51]–[55]. However, there were no significant SNPs associated with zinc accumulation within 200 kb for any of these genes.

For copper, there is evidence that a number of the *COPT* genes (*COPT*1, 2, 3, 4, 5, 6 and 7) transport copper in rice [56], [57]. *COPT1* (LOC_Os01g56420) and *COPT2* (LOC_Os01g56430) are neighbouring genes on rice chromosome 1 at 32523328–32524183 bp and 32526290–32526942 bp, respectively. For *COPT1* and *COPT2* there were a large number of SNPs significantly associated with grain copper concentration in the analysis conducted for the *tropical japonica* subpopulation at the Arkansas field site in 2006 (Figure 2). There were no other significant SNPs associated with grain copper located within 200 kb of the other *COPT* genes.

### Lack of Co-localisation for Quantitative Trait Loci

One of the most striking results of this study was the lack of consistency of the detection of associations across multiple sites. Some stability of QTLs across years may have been expected as it has been demonstrated that for the traits measured in this study there is high genetic broadsense heritability [17], [21], [22] and in this study the variation explained by genetic variation is between 20–74% (Table 1). Using the conventional approach of GWA mapping (using a P-value and MAF cut off) no associations were detected in more than two field sites at any one genomic location (Figures 1–4). This improved slightly when using the SNP enrichment method, where a number of SNPs were detected in four or five field experiments (Table 4). However, this general lack of co-localisation of significant associations across multiple sites indicates the large effect that the environmental conditions had on the underlying genetics. Previously Zhao et al. [5] observed the effect that different environments can have on the flowering time GWA mapping. It may not be surprising that the environment will have such a large effect on the grain accumulation of elements from the soil, like arsenic which is discussed below as an example.

Arsenic is not an essential element for plants so it is unlikely that a mechanism has evolved specifically for its uptake. Furthermore, arsenic’s bioavailability in the environment is complex. A relationship between soil arsenic concentration and accumulation in grains has been demonstrated but is not particularly strong and in some cases not linear [58], [59]. Also, under anaerobic conditions arsenic is mainly present as arsenite while under aerobic conditions it is present as arsenate [60]. This is important in terms of bioavailability at the time of flowering and grain loading, as different cultivars will flower at different times, and if the soil conditions are different (especially in relation to redox state) this could affect the species of arsenic which is bioavailable and which uptake mechanism it would be utilising. It was shown in a number of the field sites, for the cultivars used in this study, that there were significant differences for grain arsenic when the cultivars were grouped based on flowering time [35]. Flowering time has also been shown to have an effect on the mapping of arsenic QTLs. In the study by Norton et al. [17] large effect arsenic QTLs (which had previously co-localised with flowering time QTLs) were not detectable when the data was corrected for variations in flowering time. It has also been demonstrated that environment greatly affects the accumulation of arsenic [31], [34], [35]. Additionally, it has been demonstrated that there is a genotype by environment interaction for the accumulation of arsenic [31], so having different genomic regions being significant in different environments may be related to differing environmental adaptation of the cultivars. Research evaluating the importance of flowering time to the genotype by environment interaction for grain elements is required.

The most likely environmental factors that might explain QTL × environment interactions include climate, day length (as it impacts flowering) and soil chemistry. Mean monthly maximum and minimum temperatures for the four field sites during the experimental period are given in Table S2. The monthly averages for daily high and low temperatures during the Texas 2009 season (planted 5/5/2009) were, on average, 5°C warmer than those experienced in the two Arkansas seasons, while the temperatures during the Faridpur, Bangladesh winter (dry) growing season, and the Qiyang, China 2009 summer season were approximately 3 and 8°C cooler than those of the Arkansas, growing season, respectively. In the US sites, complete data is available for temperature allowing a comparison between Arkansas experiments in 2006 and 2007 (data not shown). While the monthly averages determined from daily high and low temperatures for the primary rice growing season (May – October) in Arkansas were nearly identical between 2006 and 2007, there was a cool spell one week after planting in 2007, while the 2006 planting was followed by warm temperatures, resulting in slowed growth of the 2007 seedlings ultimately reflected in the fact that heading dates were, on average, 2 weeks later in 2007 than in 2006 in spite of the 4-days earlier planting. The observations above might suggest temperature has a strong role in determining plant growth and ultimately grain element composition but with so little data (in terms of sites) validation or quantification are, as yet, impossible.

Another factor that could also affect the detection of QTLs across multiple years is the different experimental setups that were used. The field management was performed differently for each site, generally reflecting the common practises (except the Texas field site) of rice cultivation in the region. The Texas field site was kept flooded until harvest, a practise not carried out at the other field sites. These differences in field management may have contributed to the lack of consistency of QTLs across field sites.

### Considerations for Genome Wide Association Studies in Rice

A potential limitation with the analysis conducted here is the small number of genetic markers at present, a total of 36,901 across the genome, which equates to 1 marker every ∼11.7 kb. This becomes more of an issue when looking at the number of markers within the subpopulations as not all markers are informative (polymorphic) in all subpopulations. For example, the *temperate japonica* subpopulation analysis only uses 13,295 SNPs which equates to 1 SNP every ∼32.2 kb. Therefore, the addition of more markers to this population in the near future with a high density rice array (SR McCouch, personal communication June 26, 2013) should improve the resolution at which mapping could be done. The population used in this study is an excellent germplasm collection representing the wide geographical and ecological diversity of rice [7]. This diversity means that a large number of haplotypes will be present, but a method has to be adopted to remove the population structure, in this case a mixed model approach. The study conducted by Huang et al [3] overcame part of the problem of population structure by conducting GWA mapping using only *indica* cultivars. Using populations with high levels of genetic diversity and those with less population structure has advantages and disadvantages. Zhao et al. [5] highlighted some of these issues when using a diverse population and a mixed model mapping approach; when alleles segregate in only one subpopulation (e.g., *temperate japonica*) the mixed model approach will miss these associations, however if the alleles segregate across multiple subpopulations the mixed model has the best power to identify them. The approach of using a single subpopulation [3] is limited in that it does not include as much genetic diversity as a population containing all the subgroups of rice, however, using a single population does allow a large population with little or no structure to be analysed. For rice, other local populations are being developed; this includes an *aus* population where the accessions have been collected from Bangladesh and India [61]. For *A. thaliana* a regional population (RegMap) has been developed for GWA mapping [62]. This panel comprises of 1307 accessions with known geographic locations for ∼1200 accessions [63]. Using this population of *A. thaliana,* GWA mapping within both global and regional populations can be performed, which allows for the comparison of the genetic basis of adaptation of traits within ecologically different subsets [62]. If the number of accessions within the rice diversity panel were increased a similar approach could be adopted in rice.

The data presented here have been analysed using the standard methods for this population [4], [5]. Recently Korte et al. [64] developed a method called multi-trait mixed model (MTMM), which is a mixed model approach using correlated traits. This method can be used on the data set produced here in two different ways: firstly it could be used to look at the same trait across multiple environments to identify genes that are involved in differential responses to the environment. Secondly, as a number of elemental concentrations are correlated within rice grains [22], [65], by using more phenotype data (for example grain iron, cadmium, selenium, magnesium and manganese concentrations) it is possible to increase the power of the GWA studies to identify common associations.

### Summary

GWA mapping of grain composition for four elements in rice plants grown in five experiments has revealed associations with a complex pattern of QTL × environment interaction. Despite this complexity a number of loci and candidate genes have been highlighted by the GWA mapping for future research. These include loci where QTLs have previously been detected for arsenic, copper, molybdenum and zinc (Figures 1–4), and loci close to known transporters of copper (COPT1 and COPT2) as well as orthologues of molybdenum transporters (MOT1, MOT2, and SHST1).

## Materials and Methods

### Rice Mapping Population

The cultivars used in this study were from an established rice diversity panel [5], [7]–[9]. The RDP1 panel consists of 421 *O. sativa* cultivars collected from 79 countries, and has representatives from each of the major rice subpopulations (*indica*, *aus*, *tropical japonica, temperate japonica* and *aromatic* (Group V)) [5]. The cultivars were genotyped using 44,000 SNPs leading to the identification of 36,901 high-performing SNPs [5].

### Field Experiment

The experimental design and rice growth conditions have previously been described in Zhao et al. [5] and Norton et al. [35]. A total of 312 *O. sativa* cultivars were grown at the Faridpur field site (in a farmer’s field where permission had been granted) and at the Qiyang field site (Red Soil Experimental Station) 295 were grown. At both these sites the plants were transplanted in a randomised complete block design (RCBD) with four replicates. Plants were hand transplanted and each replicate consisted of 10 hills (one plant per hill) 20 cm apart. Each row was sown 20 cm apart from the previous, and every other row was a check variety. All the grains from all of the panicles from the six central plants from each row were harvested by hand, and a subsample of the grain was dehusked for elemental analysis.

For the field site in Arkansas (University of Arkansas Rice Research and Extension Center), the field layout in both years was a RCBD with two replications. In 2006 336 cultivars were sown in to the field and in 2007 348 cultivars were sown. Seeds of each cultivar were planted with a drill seeder about 2 cm deep in a single row 5 m long with spacing of 25 cm between each plant and 50 cm between the rows. Three representative plants were harvested by hand for the grain elemental determination.

For the field site in Texas (Texas A&M AgriLife Research Center), 370 cultivars were grown using a RCBD with three replications. Plots were planted using the same methods as those used in Arkansas. Five seeds per cultivar were drill-seeded ∼2 cm deep into 13 cm length lines, hereafter called hills. Five hills were planted per row with 61 cm between hills within each field-row, and 25 cm between rows. Genotypes were represented by one hill per replication. Twenty fully mature seeds per hill were dehusked, from which three seeds were randomly selected for elemental analysis. The fields were fertilized in accordance with standard production systems in each growing area (see Norton et al. [35] for more details of soil fertility and amendments), and flooded in the seedling stage. In Texas, the flood was maintained until all plots were mature and harvested by hand. In all other locations, fields were drained prior to harvest.

### Grain Element Composition Analysis

The methods for total element analysis were different as the optimised standard procedures at the institutes where the samples were analysed differed. The Bangladesh and China samples were analysed at the University of Aberdeen, UK, whereas the Texas and Arkansas samples were analysed at Purdue University, USA.

The following method was used for the analysis of the Faridpur and Qiyang samples. Trace-element grade reagents were used for all digests, and for quality control replicates of certified reference material (CRM) (Rice flour (NIST 1568a)) were used; spikes and blanks were included. Rice grain samples were dehusked, oven dried (80°C), and 0.2 g weighed into 50 ml polyethylene centrifuge tubes. Samples were microwave digested with concentrated HNO_3_ and H_2_O_2_ as described in Sun et al. [66]. Total elemental analysis was performed by Inductively Coupled Plasma Mass Spectrometry (ICP-MS) (Agilent Technologies 7500). Rhodium (10 µg L^−1^) was run on an external line as the internal standard. Analysis was performed as described in Sun et al. [66].

The following method was used for the analysis of the grain from the Texas and Arkansas sites. Three whole grains of fully mature dehusked rice (∼0.05 g) were digested with 1 ml of concentrated HNO_3_ in 16×100 mm Pyrex tubes, at temperatures stepped from ambient to 110°C over a period of 12 h. Indium (EM Science) was added to the acid to a final concentration of 20 µg L^−1^ as an internal standard. Samples were diluted to 10 ml and analyzed on a PerkinElmer Elan DRCe ICP-MS for total element analysis. Portions of the samples were combined and used as a matrix-matched standard for drift correction, measured after every nine samples. Samples were normalized to the averaged signals of the best-measured elements and weights of seven samples per run.

### Genome Wide Association Mapping Statistical Analysis

GWA mapping was performed using a mixed model on all the cultivars (including the admix and aromatic cultivars), and also for each of the four subpopulations separately according to Zhao et al [5]. Briefly, a mixed effects model was used to model the association between SNPs and each phenotype whilst accounting for population structure using the R package EMMA [67]. Information about population structure [5] was incorporated into models of all cultivars as both fixed and random effects whereas for models of each of the four subpopulations random effects only were used. For the fixed effects, population structure was included as the first four principal components of a principal components analysis of all SNPs across cultivars [68]. Random effects were estimated by using a kinship matrix [5] which measured the genetic similarity between individuals as the proportion of times a given pair of cultivars had the same genotype across all SNPs (IBS values).

Two approaches were used to identify if there were any genomic regions significantly associated with a trait and present in more than one GWA mapping analysis. The first method looked at the regions with significant SNPs (where a SNP was determined to be significant if the P-value was <0.0001 and the MAF >5%). In previous studies [4], [69] the level of significance was determined based on the a priori knowledge of candidate genes for the traits. This information was used to set a level of significance for which there was an enrichment of significant genomic regions containing these candidate genes. However, for most of the traits described here, there is little previous knowledge of candidate genes, therefore an arbitrary value of P<0.0001 was set for the significant threshold in this study. Chromosomal regions from different analyses (including the within subpopulation analyses) were determined to be co-localised if the significant SNPs fell within 200 kb of each other. For many traits previously analyzed in the RDP1, the maximum-effect locus fell within a 200 kb region containing the previously identified candidate genes [5]. For the grain arsenic data across all analysis (all the subpopulation GWA analysis and the “all” GWA analysis, across all five sites) the association of SNPs with the traits was explored using two other p-thresholds (using the same MAF cut-off). Initially, 330 SNPs were identified as significant (i.e. had a p-value lower than 0.0001). When the data was corrected using a Bonferroni correction of the 330 SNPs, 39 of these were below the threshold. When a Benjamini Hochberg correction was applied to the data, 236 of the 330 SNPs were significant at the 5% level.

The second method used a SNP enrichment approach. For this the top 1% most significant SNPs only were analysed (regardless of MAF) from the GWA mapping that used all the data per element (i.e., this was not done on the individual subpopulations) for each field experiment. These SNPs were then analysed to determine if they were present in multiple experiments.

### Identification of Rice Orthologues for Candidate Genes

Protein sequences of known transporters of molybdenum from different species were compared to rice protein sequences using BLASTp. The default parameters were used for BLASTp with the species specified as *Oryza sativa*.

The protein sequence of the four known transporter genes of molybdenum in plants were obtained from the National Center for Biotechnology Information (NCBI) database. The closest rice orthologue(s) for these four known transporter genes were identified using BLASTp analysis against the RGAP. The closest rice orthologue to the *A. thaliana MOT1* gene (NP_180139) [41] is located on chromosome 8 at 86335–88510 bp (LOC_Os08g01120) (E-value 1.8e^−142^). There are at least three rice orthologues for *SHST1* from *S. hamata* (CAA57710) [47]; one is located on chromosome 3 at 4984577–4992411 bp (LOC_Os03g09970) (E-value 2.5e^−239^), another on chromosome 8 at 19427423–19432708 bp (LOC_Os08g31410) (E-value 2.7e^−233^), and the third is located on chromosome 3 at 4996773–5002177 bp (LOC_Os03g09980) (E-value 3.1e^−218^). For *MOT2* from *A. thaliana* (NP_178147) [48] there is a single rice orthologue on chromosome 1 at 26034930-26033145 bp (LOC_Os01g45830) (E-value 7.3e^−146^). For *MOT2* from *C. reinhardtii* (AEY68285) [49] there are two possible rice orthologues: one on chromosome 10 at 20091716–20095059 bp (LOC_Os10g37520) (E-value 6.9e^−128^) and the other on chromosome 3 at 842386–846445 bp (LOC_Os03g02380) (E-value 3.4e^−126^).

## Supporting Information

Figure S1 **Correlation for elements at the Arkansas field site across both years.** (A) copper P<0.001, r = 0.407; (B) zinc P<0.001, r = 0.630; (C) arsenic P<0.001, r = 0.407; (D) molybdenum P<0.001, r = 0.555.
(TIF)

Table S1 **Elemental concentrations within each subpopulation at the five field experiments.**
(DOCX)

Table S2 **Mean monthly temperatures (°C) and total rainfall for the regions where field experiments were conducted (from** www.worldweatheronline.com **).**
(DOCX)

Table S3 **List of significant SNPs associated with the accumulation of arsenic within rice grains.**
(XLSX)

Table S4 **List of significant SNPs associated with the accumulation of copper within rice grains.**
(XLSX)

Table S5 **List of significant SNPs associated with the accumulation of molybdenum within rice grains.**
(XLSX)

Table S6 **List of significant SNPs associated with the accumulation of zinc within rice grains.**
(XLSX)
