# Supplementary material for: Genome Wide Association Mapping of Grain Arsenic, Copper, Molybdenum and Zinc in Rice (Oryza sativa L.) Grown at Four International Field Sites
Source: PLoS One. 2014 Feb 25;9(2):e89685. doi: 10.1371/journal.pone.0089685 (PMC3934919; doi:10.1371/journal.pone.0089685)
Supplement: Table S1 — Elemental concentrations within each subpopulation at the five field experiments. (DOCX) [file pone.0089685.s002.docx]

| Site | Trait | *Aus* | | *Indica* | | *Temperate japonica* | | *Tropical japonica* | |
| --- | --- | --- | --- | --- | --- | --- | --- | --- | --- |
|  |  | N | Mean (± SD) | N | Mean (± SD) | N | Mean (± SD) | N | Mean (± SD) |
| Faridpur | Grain As (mg kg^-1^)# | 51 | 0.428 (0.141) | 53 | 0.509 (0.125) | 69 | 0.392 (0.097) | 79 | 0.471 (0.095) |
| Faridpur | Grain Cu (mg kg^-1^) | 51 | 3.78 (0.67) | 53 | 3.36 (0.63) | 69 | 4.55 (0.77) | 79 | 3.37 (0.57) |
| Faridpur | Grain Mo (mg kg^-1^) | 51 | 0.941 (0.140) | 53 | 0.897 (0.211) | 69 | 1.067 (0.278) | 79 | 1.049 (0.220) |
| Faridpur | Grain Zn (mg kg^-1^) | 51 | 18.7 (4.1) | 53 | 14.8 (2.8) | 69 | 19.3 (3.4) | 79 | 15.1 (2.6) |
| Qiyang | Grain As (mg kg^-1^)# | 51 | 0.765 (0.146) | 48 | 0.754 (0.140) | 61 | 0.709 (0.134) | 77 | 0.547 (0.097) |
| Qiyang | Grain Cu (mg kg^-1^) | 51 | 0.573 (0.203) | 48 | 0.810 (0.426) | 61 | 0.916 (0.304) | 77 | 0.737 (0.500) |
| Qiyang | Grain Mo (mg kg^-1^) | 51 | 1.42 (0.26) | 48 | 1.39 (0.47) | 61 | 1.51 (0.38) | 77 | 1.72 (0.31) |
| Qiyang | Grain Zn (mg kg^-1^) | 51 | 18.7 (3.7) | 48 | 16.0 (3.7) | 61 | 18.0 (3.3) | 77 | 14.2 (4.0) |
| Ark2006 | Grain As (mg kg^-1^)# | 55 | 0.463 (0.140) | 57 | 0.413 (0.147) | 79 | 0.318 (0.096) | 83 | 0.377 (0.129) |
| Ark2006 | Grain Cu (mg kg^-1^) | 55 | 1.85 (0.39) | 57 | 2.06 (0.60) | 79 | 2.56 (0.54) | 83 | 2.32 (0.59) |
| Ark2006 | Grain Mo (mg kg^-1^) | 55 | 0.772 (0.123) | 57 | 0.728 (0.149) | 79 | 0.605 (0.138) | 83 | 0.710 (0.117) |
| Ark2006 | Grain Zn (mg kg^-1^) | 55 | 26.5 (2.8) | 57 | 22.0 (2.1) | 79 | 26.8 (2.3) | 83 | 25.5 (2.3) |
| Ark2007 | Grain As (mg kg^-1^)# | 54 | 0.396 (0.225) | 62 | 0.264 (0.189) | 87 | 0.221 (0.151) | 80 | 0.214 (0.098) |
| Ark2007 | Grain Cu (mg kg^-1^) | 54 | 3.20 (0.61) | 62 | 3.37 (0.70) | 87 | 4.02 (0.65) | 80 | 4.12 (0.77) |
| Ark2007 | Grain Mo (mg kg^-1^) | 54 | 0.659 (0.181) | 62 | 0.408 (0.135) | 87 | 0.382 (0.121) | 80 | 0.441 (0.115) |
| Ark2007 | Grain Zn (mg kg^-1^) | 54 | 31.2 (3.6) | 62 | 26.2 (2.9) | 87 | 31.9 (3.5) | 80 | 30.7 (2.7) |
| Tx2009 | Grain As (mg kg^-1^)# | 55 | 0.699 (0.215) | 71 | 0.655 (0.242) | 94 | 0.522 (0.164) | 84 | 0.651 (0.198) |
| Tx2009 | Grain Cu (mg kg^-1^) | 55 | 2.54 (0.42) | 71 | 2.66 (0.58) | 94 | 3.54 (0.55) | 84 | 3.06 (0.46) |
| Tx2009 | Grain Mo (mg kg^-1^) | 55 | 0.381 (0.100) | 71 | 0.288 (0.079) | 94 | 0.327 (0.068) | 84 | 0.379 (0.069) |
| Tx2009 | Grain Zn (mg kg^-1^) | 55 | 21.0 (3.4) | 71 | 19.3 (3.3) | 94 | 22.8 (3.2) | 84 | 22.1 (2.1) |
