# Supplementary material for: Genome Wide Association Mapping of Grain Arsenic, Copper, Molybdenum and Zinc in Rice (Oryza sativa L.) Grown at Four International Field Sites
Source: PLoS One. 2014 Feb 25;9(2):e89685. doi: 10.1371/journal.pone.0089685 (PMC3934919; doi:10.1371/journal.pone.0089685)
Supplement: Table S2 — Mean monthly temperatures (°C) and total rainfall for the regions where field experiments were conducted (from www.worldweatheronline.com ). (DOCX) [file pone.0089685.s003.docx]

Supplementary Table 2. Mean monthly temperatures (^o^C) and total rainfall for the regions where field experiments were conducted (from [www.worldweatheronline.com](http://www.worldweatheronline.com)).

| Month | Faridpur, Bangladesh | | Qiyang, China | | Beaumont, Texas | | Stuttgart, Arkansas | |
| --- | --- | --- | --- | --- | --- | --- | --- | --- |
|  | Mean max. | Mean min. | Mean max. | Mean min. | Mean max. | Mean min. | Mean max. | Mean min. |
| December | 25 | 12 |  |  |  |  |  |  |
| January | 23 | 10 |  |  |  |  |  |  |
| February | 26 | 12 |  |  |  |  |  |  |
| March | 29 | 14 |  |  |  |  |  |  |
| April | 31 | 19 | 15 | 12 | 26 | 14 | 24 | 12 |
| May | 29 | 19 | 21 | 17 | 29 | 19 | 28 | 17 |
| June |  |  | 23 | 21 | 32 | 22 | 32 | 21 |
| July |  |  | 27 | 24 | 33 | 23 | 34 | 23 |
| August |  |  | 28 | 24 | 33 | 23 | 34 | 22 |
| September |  |  |  |  | 32 | 21 | 30 | 18 |
| October |  |  |  |  | 28 | 15 | 24 | 12 |
| **Rainfall during experiments** | | | | | | | | |
|  | No rain Dec-May | | 12 cm per month | | 20 cm April, 12 cm per month May-October | | 15 cm April, 9 cm per month May-October | |
